# Tapping the Power of Soda Taxes: A Call for Multidisciplinary Research and Broad-Based Advocacy Coalitions – A Response to the Recent Commentaries

**DOI:** 10.15171/ijhpm.2018.30

**Published:** 2018-04-08

**Authors:** Sarah A. Roache, Lawrence O. Gostin

**Affiliations:** O’Neill Institute for National and Global Health Law, Georgetown University Law Center, Washington, DC, USA.

## Introduction

In comments on our recent editorial, Le Bodo and De Wals^1^ and Baker et al^2^ provide compelling reflections on the need for further research into the policy processes and societal conditions conducive to sustainable soda taxes. This response is a call to action for increased multidisciplinary research and broad-based advocacy coalitions to expand the use and the effectiveness of soda taxes to promote the public’s health. In particular, we highlight the need for research relevant to low- and middle-income countries (LMICs) and emerging efforts to incorporate the voices and experiences of people living with non-communicable diseases (NCDs) into the development of policy responses.

### A Multidisciplinary Research Agenda on Public Health-Based Soda Taxes

Soda taxes have been adopted in more than 25 jurisdictions across the world,^2^ providing a growing body of data to evaluate and improve existing and future interventions. To date, most research on soda taxes has focused on potential and actual impacts on soda consumption patterns, especially among low-income groups.^3,4^

Drawing on case studies and political science theories, Baker et al identify conditions conducive to the adoption of sustainable soda taxes, including fiscal need, anticipating and countering industry opposition, and framing the revenue raising and public health benefits of soda taxes to generate public support.^2^ Le Bodo and De Wals call for the expansion of theory-driven research to further elucidate feasibility and acceptability, highlighting Sabatier’s advocacy coalition framework and Kingdon’s multiple streams theory as particularly useful in analyzing obesity prevention policy processes.^1^ In some jurisdictions, soda taxes have been proposed and rejected (eg, Colombia,^5^ Santa Fe, New Mexico^6^) and in many others, they are the subject of ongoing debate (eg, Australia,^7^ Canada,^8^ Singapore^9^). Research grounded in theoretical frameworks of social change promise valuable insights to determine how advocates and policy-makers might overcome barriers to adoption.

As soda taxes are a relatively new phenomenon, further future research will be required to quantify the impacts of soda taxes on bodyweight and disease.^10^ A robust research agenda will also address tax-related industry reformulation, product substitution by consumers in response to price increases, and the health impacts of alternative products, such as artificially sweetened drinks. As Le Bodo and De Wals note, it will incorporate analysis of optimal tax design, including whether taxes apply to non-caloric sweetened beverages and whether they are structured as specific excise taxes or ad valorem taxes.^1^ In light of industry litigation challenging soda taxes in Philadelphia, Pennsylvania,^11^ and Cook County, Illinois,^12^ analysis of the legal grounds and arguments is warranted. A robust research agenda will help foster public demand and political will in support of new taxes, optimize tax design and implementation of existing taxes, and ensure they can withstand industry opposition and legal challenges.

The breadth of issues described above shows the importance of a multidisciplinary approach to research on soda taxes. Relevant disciplines include public health, epidemiology, behavioral science, economics, political science, and law. As the soda industry increasing expands markets LMICs,^13^ it is crucial that research considers and is adapted, insofar as is possible, to different economic and sociocultural contexts.^14^ Relevant research questions include whether taxes can help address the dual burdens of under- and over-nutrition^15^ and how to avoid soda taxes leading to decreased fluid intake among populations with limited access to safe drinking water.^16^ Experts in development and implementation science can help address challenges facing LMICs considering soda taxes, though engagement of local experts is crucial to ensure relevance and sustainability.

### Building Stronger Advocacy Through Coalitions

Advocacy among local organizations, philanthropists, lobbyists, politicians, and celebrities has played a key role in the adoption of many existing soda taxes, including in Mexico,^17^ the United Kingdom,^18^ and cities and counties throughout the United States.^19^ Effective advocacy efforts can seize on favorable political windows (eg, the coalescence of budgetary deficits and alarming rates of disease) to promote adoption of soda taxes^1^ or help foster favorable societal conditions by raising awareness and generating public support.

Baker et al highlight the effectiveness of a broad-based advocacy coalition in Mexico, which comprised an alliance of local organizations, universities, and lobbyists, and drew on technical support from the Pan American Health Organization (PAHO) and financial support from Bloomberg Philanthropies.^2^ In Barbados, academics, health promotion advocacy groups, and PAHO are working together to protect that country’s 10% excise tax on sugary drinks, which is the subject of an industry campaign for repeal.^20^ These types of coalitions show the power of the engagement of local, national, and international actors from a broad range of sectors.

People living with NCDs are an important but underrepresented constituency in conversations about promoting healthier diets. Traditionally, NCDs and their risk factors have been framed as an issue of individual responsibility, and people living with NCDs have not had a strong collective voice in advocacy for prevention and care. In February 2018, the NCD Alliance, a global network of civil society organizations working to combat the NCD epidemic, released an *Advocacy Agenda of People Living with NCDs*.^21^ The agenda calls for a range of prevention, treatment, and support measures, including taxes on harmful and unhealthy products and the inclusion of people living with NCDs in the development of policy responses. Multisectoral coalitions, especially those incorporating the voices and experiences of people living with NCDs, offer potential to negate the industry-driven narrative of individual responsibility in favor of collective health promotion strategies.

## Conclusion

A robust multidisciplinary research agenda, addressing policy processes, design, implementation, and impacts, has the potential to accelerate adoption and maximize the public health and social benefits of soda taxes throughout the world. Broad-based advocacy coalitions also contribute to this goal, offering benefits of diverse experiences and strategies, financial and technical resources, and enhanced leverage among policy-makers. Local and global actors – people living with NCDs, academics, philanthropists, politicians, among many others – can help tap the power of soda taxes to improve public health.

## Ethical issues

Not applicable.

## Competing interests

Authors declare that they have no competing interests.

## Authors’ contributions

SRA wrote the first draft of the manuscript with inputs from LOG. Both authors contributed to iterations of the manuscript and approved the final version.
